# 

**Published:** 1891-10

**Authors:** 


VOL. XII.] *PUBLISHED MONTHLY AT THREE DOLLARS A YEAR. SINGLE COPIES, THIRTY CENTS.* [No. 10.

Entered at the Post Office, February 3rd, 1891, at New York as Second-class Matter.
Copyright, 1891, by W. A. Conklin, and R. S. Huidekoper.

### THE JOURNAL
OF
### COMPARATIVE MEDICINE
AND
### VETERINARY ARCHIVES.

EDITED BY

W. A. CONKLIN, Ph. D., D. V. S.,
*Director of Zoological Gardens, New York.*

RUSH SHIPPEN HUIDEKOPER, M. D., Veterinarian, (*Alfort.*)
*Professor of Sanitary Medicine and Veterinary Jurisprudence,*
*American Veterinary College,*
*New York.*

OCTOBER, 1891.

All communications and payments should be addressed to

**MANAGING EDITOR JOURNAL COMP. MED. AND VET. ARCH.**

FORTY-SECOND STREET AND PARK AVENUE, NEW YORK.

Printed for the Editors by

WILLIAM R. JENKINS, PUBLISHER,

NEW YORK.

**LONDON: BALLIERE, TINDALL & COX.**

